# Empirical vitalism: observing an organism’s formative power within an active and co-constitutive relation between subject and object

**DOI:** 10.1007/s40656-024-00649-z

**Published:** 2025-01-24

**Authors:** Christoph J. Hueck

**Affiliations:** Akanthos Academy, Zur Uhlandshöhe 10, 70188 Stuttgart, Germany

**Keywords:** Immanuel Kant, Autopoiesis, Objectification, Organic development, Participatory science, Goethe’s morphology

## Abstract

This article proposes an empirical approach to understanding the life of an organism that overcomes reductionist and dualist conceptions. The approach is based on Immanuel Kant’s analysis of the cognitive conditions required for the recognition of an organism: the concept of teleology and the assumption of a formative power of self-generation. It is analyzed how these two criteria are applied in the cognition of a developing organism. Using the example of a developmental series of a plant leaf, an active and relational process between observer and developing organism is shown, within which the teleology and self-generating power of the organism can be empirically observed through the mental faculties of understanding and will. Furthermore, it is emphasized that, according to Kant, even physical objects are not readily given, but are actively constituted through the unification of sense perceptions with concepts. This Kantian mode of objectification facilitates cognition of the physical properties of an organism. It can be supplemented with a participatory and co-constitutive mode of realization, in which the teleologically organizing and self-generating power of the organism can become an object of empirical research. It is argued that the participatory mode also facilitates an expanded conception of nature that allows for the existence of living beings within it. Finally, an analogy to Goethe’s approach to the living organism is highlighted. In summary, it is stated that it is possible to understand life by consciously participating in it.

## Introduction

Living organisms pose a perennial problem to scientific understanding: They are material entities and as such conform to physical laws, but they act purposefully as if they were intelligent beings. How can matter act in an intelligent way?—During the second half of the twentieth century, this problem was thought to be solved. It was assumed that self-replicating entities initially emerged from complex prebiotic chemistry (Pross, 2016) and further evolved in a random and statistically selected way as genetically programmed survival machines (Mayr & Provine, 1980; Dawkins [1976], 2009). In recent decades, however, this conviction has been increasingly challenged (Strohman, 1997; Moss, 2004; Nagel, 2012; Laland et al., 2015; Sultan et al., 2021), and there is an ongoing debate about complementary views in both biology and philosophy (Rosslenbroich, 2014; Laland et al., 2015; Walsh, 2015; Noble, 2016; Nicholson & Dupré, 2018; Walsh, 2018; Gambarotto & Mossio, 2022; Gambarotto & Nahas, 2023; Walsh & Rupik, 2023; Rupik, 2024; Wolfe, 2024).^1^ A sophisticated theory of the self-generation of organisms (autopoiesis) has been developed (Luisi, 2003; Maturana & Varela, 1980) which describes living entities as autonomous, self-generating, teleological and adaptive agents (Di Paolo, 2005; Fábregas-Tejeda et al., 2024; Kauffman & Clayton, 2006; Moreno & Mossio, 2015; Mossio, 2024; Nicholson, 2013; Okasha, 2024; Švorcová, 2024). However, although this theory focuses on the living organism itself (and does not lose sight of it between genetic reductionism and population statistics), it is merely descriptive, but does not explain the ontological status of organisms (Wolfe, 2010, 2024). Organisms are natural beings, but if nature is conceived of in mere materialistic and mechanistic terms, it is difficult to find a place in it for self-generation, teleology and autonomous agency.^2^

Alternative approaches to solve the organism-problem are vitalistic theories. “Vitalists hold that living organisms are fundamentally different from non-living entities because they contain some non-physical element or are governed by different principles than are inanimate things” (Bechtel & Richardson, 1998, p. 1). Such non-physical elements are usually considered as “obscure supernatural agencies acting within organisms” (Nicholson & Gawne, 2015, p. 347). Therefore, vitalism is generally dismissed as unscientific, although it may be granted a certain heuristic value for the description of organisms (Donohue & Wolfe, 2023) and even considered to enable logical deduction of concepts and laws of the living organism (Chen, 2024). However, the general problem of vitalistic approaches is that “non-physical elements” in organisms cannot be empirically observed and that vitalism, therefore, remains a theoretical approach that cannot be confirmed by experience.

The organism-problem was already fundamentally analyzed by Immanuel Kant in his *Critique of Judgment* (Kant [1790], 2008, AA 5). Kant focused on the gap between the necessity to describe organisms as teleological and self-generating entities on the one hand, and the impossibility to explain these properties in naturalistic terms on the other, and his analysis thus fits perfectly to the current debate. The lasting importance of Kant’s analysis can be seen in the fact that he went to the root of the problem, namely the *conditions of knowability*, i.e., how we must conceive of an organism.^3^ He showed that an organism can only be understood if (i) each of its parts is seen as teleologically linked to the others and to the whole (ibid., 5:373) and (ii) that it generates and maintains itself.^4^ Teleology and self-generation, however, cannot be viewed as properties of (material) nature, since they presuppose (i) the idea of the whole (ibid.) and (ii) of a producing causality of “acting according to ends” (ibid., 5:389). However, as Kant stated, “the universal idea of nature, as the sum of objects of the senses, gives us no reason whatever for assuming that things of nature serve one another as means to ends. (…) [For] we do not take [nature] to be an intelligent being” (ibid., 5:359).

Objects of the senses are material, and matter is inert, while organisms change “from an internal principle” (Kant [1786], 2004, AA 4:544).^5^ Kant thus claimed that although organisms must be *described* in terms of teleology and self-generation, they cannot be *explained* as such, that is, their essential properties cannot be derived from material and mechanical causes (Kant [1790], 2008, p. 5:418). The subjectively necessary interpretation of organisms as teleologically self-generating entities cannot be unified with an objective, mechanistic and materialistic conception of nature.^6^

Here, I start from Kant’s analysis and show its significance for the organism-problem (Sect. 2), but do not stop with it. Instead, I suggest a solution to the organism-problem that lies in adopting *a different cognitive relationship* to the teleologically self-generating organism then to merely physical objects. I demonstrate this relation with the example of a developmental series (Sect. 3). In Sect. 4, I describe the subject-object relationship as more active and participatory in the cognition of an organism than in the cognition of physical objects. I argue that such a way of knowing facilitates an outlook on an expanded conception of nature (Sect. 5) and that it enables an empirical approach to the teleological self-generating power of organisms (Sect. 6). I conclude by discussing some aspects of this approach and relating it to Goethe’s approach to the organism.

## Significance of Kant’s analysis of the organism-problem

In the *Critique of Judgment* Kant showed that in order to cognize an organism, one must apply “concepts of reason” and assume a productive power capable of “acting according to ends”, just as one must accept concept-guided, purposive action as the cause of a hexagon drawn on the sand (Kant [1790], 2008, 5:370). Importantly, Kant did not primarily discuss the objective status of an organism but the problem of subjectively cognizing it—“even to know it empirically in respect of its cause and effect” (ibid., 5:370). Organisms are certainly possible in nature, but how do we know about them? In other words: How do we know the difference between an artificial and a living plant, even if the two were sensually indistinguishable? Kant showed the necessary conditions of knowability that must be met to cognize a living organism at all. This epistemological turn has important implications for the philosophy of biology, because it shows that it is simply not possible to conceive of an organism (let alone talk about and discuss it) without the concept of its teleological and self-generating wholeness.

Therefore, any attempt to interpret an organism’s apparent purposiveness in mechanical terms presupposes “an idea of what counts as evidence of purposiveness in the first place” (Hverven & Netland, 2023, p. 315). However, the necessity of applying such “concepts of reason” is not always reflected upon. It can be argued that Kant’s lasting contribution to the philosophy of biology is that he provided the argument for making this necessity conscious. Thus, even if this concept of teleological self-generation is not explicitly stated, it must be necessarily implied in any knowledge about an organism.^7^

## Knowing teleological organization and self-generation

According to Kant, the concept of teleological self-generation (“acting according to ends”) cannot be reconciled with the concept of nature “as the sum of objects of the senses”, and “the actual existence of these ends cannot be proved by experience” (Kant [1790], 2008, p. 5:359).^8^ Although this latter notion seems evident, it may be questioned. Couldn’t it be possible to attain *an experience* of the teleological forces which are active in the self-generation of an organism? According to Kant, experience of physical (dead) objects results from the unification of sensual perceptions with concepts. Couldn’t it be possible to approach a living organism in a way that would enable an experience, if not a sensory, of its teleological self-generating power?

The quest for such an empirical approach to the organism-problem has led to the proposition that we have an immediate access to the reality of life by experiencing or own strivings as a living being (Jonas [1966], 2001). Accordingly, Andreas Weber und Francisco Varela wrote that “we strive to go on, to develop, to keep ourselves in a dynamical balance” (Weber & Varela, 2002, p. 100), and suggested that “[i]t is actually by experience of *our* teleology (…) that teleology becomes a real rather than an intellectual principle” (ibid., p. 110). In addition, they proposed that the experience of “our teleology” can facilitate an understanding of the intrinsic teleology of other organisms: “[I]n observing other creatures struggling to continue their existence—starting from simple bacteria that actively swim away from a chemical repellent—we can, by our own evidence, understand teleology as the governing force of the realm of the living” (ibid.).

However, although our inner experience of being alive seems to be an indisputable fact, the theoretical transfer of this experience to other organisms is questionable. Kant had called it a “mental jugglery that only reads the conception of an end into the nature of the things” (Kant [1790], 2008, 5:359), and accordingly such transfer has been criticized as unscientific anthropomorphism (Villalobos & Ward, 2016).

**Fig. 1:**
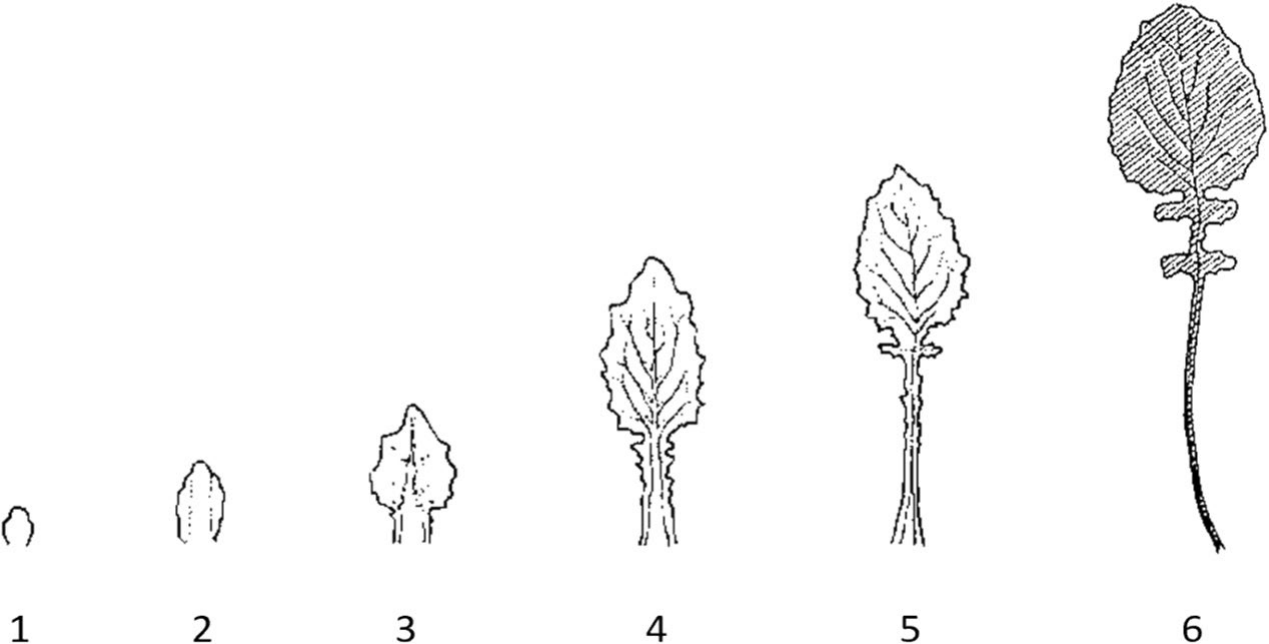
Sketch of developmental states of an individual leaf from common nipplewort (*Lapsana communis*) (adapted from Bockemühl, 1982)

An approach which searches for an experience of the teleologically self-generating force of an organism must necessarily start with the phenomena of the living organism itself. Of these, an organism’s shape and shape-changes during development are the most immediate. Therefore, organic shapes and their developmental changes are chosen here to approach the self-generating power of an organism.^9^ Consider the series of shapes shown in Fig. 1. One perceives different shapes and conceptually determines them as states of an individual plant leaf’s development which transform into each other (in the order from left to right). The continuous transformation, at least as represented in Fig. 1, is not sensually observable; it must be supplemented by the cognizing subject. While the shapes are perceived objects, the process that connects them is only created within the imagination^10^—or rather, it becomes manifest by being imaginatively created. This creation is a *willful* process which requires the focused inner activity of the observing subject. However, although being entirely dependent on this subjective activity, the process is not arbitrary. One knows exactly how to change one form to arrive at the next. For example, to move from the 5th shape to the 6th, one must expand and somewhat round the blade, enlarge the small, two-sided extension beneath the blade, add another one, and prolong, thin and somewhat bend the petiole.

The imaginative realization of these processes requires the active engagement of the observing subject. This imaginative activity is “formative”, i.e., it generates or changes forms, and—as will be discussed below—it can be related to what Kant called the “formative power” of an organism (Kant [1790], 2008, 5:374).

By supplementing the dynamic changes of the shapes, the subject *actively participates* in the developmental procedure. It consciously creates within its imagination the same shape changes as the plant does; it “re-creates” the developmental process. Without such participatory re-creation, the depicted shapes could only be identified as an organic developmental series in a very abstracted and superficial manner. The general concept of “development” does not facilitate knowledge about the detailed and gradual changes of the leaf-shapes. Thus, the *condition of knowability* of the details of organic development is that one actually re-creates them in the imagination. To put it in the words of Carl-Ernst von Baer as quoted in the epigraph, “to understand life itself, (…) it wants to be slowly observed in its manifestations, and (…) from quite different regions [than from dissecting analysis] shines the light that slowly but constantly brings these observations to greater clarity” (Von Baer, 1864, p. 82). *Slow* and re-creating observation brings organic development to more clarity.

Although the general concept of teleological development is insufficient to know the process in detail, the imaginative re-creation of the developing leaf is guided by this concept. We follow the growing leaf in the direction from the bud (1) to the fully grown leaf (6) and not vice versa.^11^ In fact, organic self-generation and teleology are inseparable,^12^ and both required for judging an organism (Ginsborg, 2015b; Huneman, 2017). Nevertheless, teleological organization and self-generation are cognized in different ways. While teleological organization is a *concept of understanding*, self-generation is related to the faculty of *will*.^13^ Kant explicitly mentioned the faculties of understanding and will and their relation to the cognition of an organism in his text *On the Use of Teleological Principles in Philosophy* (1788):

A “power that is effectuated through [an organism] has to be thought as a cause effective according to ends, and this in such a manner that these ends have to be presupposed for the possibility of the effect. But we know such powers, in terms of their ground of determination only in ourselves, namely in our *understanding* and *will*, as a cause of the possibility of certain products that are arranged entirely according to ends, namely that of works of art. In us understanding and will are basic powers, of which the latter, insofar as it is determined by the former, is a *faculty to produce something* according to an idea which is called end” (Kant [1788], 2012, AA 8:181; Kant’s emphasis omitted and my own added).

The self-generating, formative power of an organism can be related to the faculty of will, while the organism’s teleological organization relates to understanding. However, in contrast to the will that is active in creating works of art, the will with which we re-create a developmental series is not active in bodily actions, but in imagination. It is a will which is applied to and which generates dynamic mental images. When we engage in re-creation of an organic developmental process, we apply such willful activity to the representations of the different developmental states. Without this re-creation we could either only gaze at the individual shapes or have only a very abstract conception of their development and probably tend to overlook many of its intricate details. The conscious willful and participatory engagement with the complex processes of organic development, therefore, “shines a light” on these processes and brings the forces and laws that produce and govern them to greater clarity.^14^

To be sure, this willful activity is not merely subjective and surely not arbitrary. It does not dogmatically project concepts onto the developing organism, but slowly and patiently follows nature to where it leads. It could thus be called a “receiving will”, i.e., an active engagement which is being directed by the objective changes of the organism.

Is this concept-guided, willful and imaginative re-creation of a developmental series equivalent to a conscious experience of the teleologically self-generating power of an organism itself? At first sight, it appears to be a subjective activity which may be required to understand organic development, but for which Kant’s argument holds that we “borrow” it “solely from ourselves” and project it onto another being (Kant [1790], 2008, 5:361). However, Kant did not analyze what it means to empirically follow an organism in its development in a slow and patient mode, continuously adapting our mental activities to the living natural object’s change. Such mode, however, is required to observe the intimate relationship between our willful activity and an organism’s continuous development. Thus, instead of only stating *that* we relate our inner activity and experience to other beings, I have tried to describe *how* we do that. And since we would not know the detailed developmental processes of an organism without such willful engagement, it can be said that we *realize* the organism’s formative force within our mental activity. The teleological self-generation of an organism is being realized within our active encounter with it. So yes, it can be claimed that the force which we experience within this willful re-creation is at least similar to the real formative force which is active in the organism.

## Physical objectification and organic realization

As already mentioned above, Kant showed that even the cognition of physical objects is based on inner activity. The unity of an empirical “object of experience” (Kant [1788], 2012, AA 3:B126) results from an active unification of a manifoldness of sensory intuitions with concepts of the understanding.

“The combination (…) of a manifold in general can never come to us through the senses, and therefore cannot already be contained in the pure form of sensible intuition; for it is an act of the spontaneity of the power of representation, and since one must call the latter understanding, in distinction from sensibility, all combination, whether we are conscious of it or not, (…) is an action of the understanding, which we would designate with the general title synthesis in order at the same time to draw attention to the fact that we can represent nothing as combined in the object without having previously combined it ourselves, and that among all representations combination is the only one that is not given through objects but can be executed only by the subject itself, since it is an act of its self-activity” (ibid., 3:B129–B130).

Physical “objects of experience” are thus not “out there”, but are being constituted (objectified) within and dependent upon the process of cognition (Horstmann, 2018; Meer, 2018). In objectifying a physical object, one could say that we actively (but unnoticedly) place it into what appears to us as the outer “objective world”.^15^ Regarding an organism, its appearance *as a physical entity* is the result of such objectification. As a material entity, an organism appears to us in the same way as other physical objects: A certain manifoldness of sensual perceptions is being objectified by the concept “tree” in the same way as another manifoldness is being objectified by the concept “house”. If we do not go beyond *this* mode of objectification, a tree may appear to us as a purely physical object. However, what can be known in this manner is only the physical, non-living part of an organism (that which remains of it at the moment of death). Its teleological organization and self-generating power cannot be objectified in this way. According to Gertrudis Van de Vijver and colleagues, “living systems intrinsically *resist* any attempt of objectification, and demand as such for an approach qualitatively different from the one developed in relation to non-living systems” (Van de Vijver, et al., 2005, p. 58). In such an approach, according to these authors, the “directionality of the observer” and the purposiveness of the observed are reciprocally linked (ibid., p. 67). As I have shown above, such an approach is the co-constitutive mental activity which connects the observer with the organism.

**Fig. 2:**
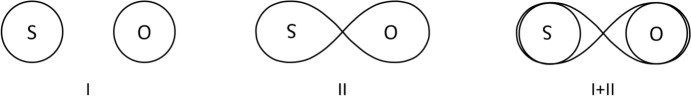
Two modes of objectification (I) and realization (II) and their combination in the cognition of living organisms. S = subject, O = object

The Kantian mode of objectification is applied to the sensually perceptible properties of an organism. In this mode, the subject experiences itself as separated from the biological object and comprehends what is physical about it (Fig. 2, left). To comprehend the life of an organism, *another mode* must be added, which is not objectification, but can be called *realization*. In this mode, the subject does not experience itself as entirely separated from the object but as actively and continuously engaged in its realization. Both the subject and the object change their status: the subject becomes more active and creative than in the cognition of physical objects, and the object becomes less passive, particular and dead. As Van de Vijver and colleagues stated, this mode of realization is “the means par excellence on the basis of which living systems (…), reveal their specificity and uniqueness” (ibid.). Therefore, cognition is something different for the physical side of an organism than it is for its life. When cognizing the living activity of an organism, subject and object are more closely related to each other, as it were; they interact (Fig. 2, middle). (To be sure, the second, relational mode does not replace the first, physical mode but must be added to it as an expansion (Fig. 2, right)).

To know the teleology and self-generating force of an organism requires to switch from a mode of physical objectification to a mode of organic, participatory realization. The knower must give up its position as a mere and largely passive observer of what appears to him as external reality, and actively and consciously delve into the dynamical flow of life. He must become mentally active, but not in an arbitrary way as in phantasy and art, but in a mode of re-creation of organismic life. Thus, to understand life, one must consciously participate in life.

Knowledge of teleological self-generation of an organism is therefore not representative like knowledge of physical objects, but *participatory*. Kant claimed that purposiveness is only a principle of reflective, not of determining judgment (Kant [1790], 2008, 5:181), and that, accordingly, we can only regard organisms “as if” they were teleological and self-generating beings, but that we cannot determine them as such. Here, I argue that there is a third way of relating to an organism which is neither determinative nor reflective, but co-constitutive.

## The ontological space of the living

The epistemological discussion of the previous sections bears some implications for the ontology of living beings. First, it emphasizes Kant’s notion that even physical objects do not exist “out there” independent from our cognition. What we experience as the physical world is always the *experienced* physical world. Therefore, what we know as “physical objects” is only possible because we provide the conditions for their knowability: “The conditions of the possibility of experience in general are at the same time conditions of the possibility of the objects of experience” 2012, Kant [1787] 1998, 3:B197). Although physical objects appear to us as readily given, their appearance, according to Kant, is the result of a subtle cognitive process of “objectification”, because “we can represent nothing as combined in the object without having previously combined it ourselves” (ibid., 3:B130).

Therefore, in a transcendental perspective, what we experience as physical nature resides within a certain *ontological space*. According to Van de Vijver and colleagues, an ontological space is a “space of conditions of possibility, within which phenomena are asked to fit” (Van de Vijver, et al., 2005, p. 60). A philosophical approach to nature, thus,

“implies, firstly, an analysis of the conditions within which the systems under study can be experienced, and secondly, an analysis of the conditions under which this experience can be systematically arranged in view of a knowledge that is objective, universal and necessary. In other words, a transcendental philosophy does more than simply working within a certain ontological space. It sets up an analysis of the ontological space itself. This space can be conceived of as a space of conditionality, of experience on the one hand, and of objective knowledge on the other hand” (ibid., p. 60–61).

The ontological space of physical nature is determined by the ability to objectify sensual perceptions with unifying concepts. The ontological space of the living, however, is different. It results from a “a continuous co-determination between two terms, the observing and the observed, the interpreter and the interpreted” (ibid., p. 71). Such continuous co-determination has been shown above with the example of cognition of a developing leaf. Thus, the ontological space of the living is not constituted by “I versus it”, but by “us”.

The Kantian stance assumes that the concept of teleological self-generation is applied to entities which reside within the ontological space of physical nature, inevitably leading to the notion “as if”. However, since knowledge about the physical world depends on the respective conditions of knowability, it may be claimed that another ontology is possible, which is not constituted by determining processes of objectification but by co-constitutive processes of realization. This claim is not speculative but results from the analysis of knowing a teleologically self-generating organism in contrast to a physical object. It must be kept in mind, though, that organisms reside in both ontological spaces. Their material appearance resides within the ontological space of the physical, but not their living activities. Instead, these activities correspond to an ontological side of the organism that is not perceivable through the senses. The life of the organism *reveals itself* only within the participatory cognitive relation between the actively engaging subject and the living object. Thus, the *reality of the living* manifests itself within a two-directional, dynamic and co-constitutive encounter between the cognizing observer and the organism.

The physical features of an organism can be analyzed according to the laws of physics and chemistry, and such analyses have led to the immensely impressive results of molecular genetics, biochemistry, physiology, immunology, neurology, etc. Nevertheless, even the most extended and detailed genetic or biochemical analysis does not provide the clue to solve what has been called the “great riddle of life” (Bertalanffy, 1933, p. 67). To solve this riddle, i.e., to explain the ontological status of organisms, requires a different approach, which is outlined here.

In summary, I propose that the teleological self-generating force of an organism is not only an epistemological requirement, but an ontological reality, though not a physical one. The recognition of this force is not the result of a dualistic attitude aimed at reviving theoretical vitalism, but rather an *empirical approach* to the reality of the self-forming processes of life, since this force can be experienced and studied as conscious willful activity within the co-constitutive mental encounter with living organisms.

The assumption that nature consists only of the sum of sensory objects, i.e., that it is only material, does not allow to think of the possibility of living beings within it. Therefore, this assumption is probably not sufficiently comprehensive. I suggest to explicitly consider an additional (or expanded) “ontological space” of nature that cannot be perceived through the senses but can be observed within the re-creating activity between organisms and observer: a woven and ever-weaving, intrinsically active and intelligent web of life.^16^

## Empirical research within the space of the living

The thesis put forward here is that the life of an organism, i.e., its self-generating, teleologically organizing power can be observed through and within the lively engagement of the cognizing human mind. In contrast to physico-chemical research, the study of life therefore requires a more active and re-creative engagement of the observer. Van de Vijver and Haeck emphasized that living organisms cannot be captured as if they “exist independently from *our doings*” (Van de Vijver & Haeck, 2024, p. 66). It is the *cognitive activity* of the knowing subject with which it supplements the *vital activity* of the organism. While the Kantian mode of objectification allows the knowing subject to remain in a comparatively passive attitude, the organismic mode of co-creative realization requires its continuing active engagement.^17^ Understanding life is not a matter of knowing about fixed objects but mentally engaging in development, processes and change and at the same time observing this activity. Although this engagement depends on the subject, it is not arbitrary, since it is guided by the natural phenomena (as shown in the discussion of Fig. 1). Thus, the conscious observation of this re-creating activity opens the possibility of empirical research into the forces, processes and teleological laws of the living (cf., e.g., Bockemühl, 1998). The demonstration of the respective phenomena of the living and the precise description of the observations made within the re-creating activity will then enable intersubjective, scientific exchange and progression.

When one perceives an organism, one only sees (touches, tastes, smells, etc.) the *products* of its living activity. Life itself cannot be perceived through the senses; it is “supersensible”. Nature, therefore, appears to be more than dead matter alone. However, although being supersensible, life is not something strange and mysterious but an everyday experience. It is nevertheless an age-old question how we can understand living beings. My analysis shows how *life itself,* in its teleologically organizing and self-generating power, can become an object of empirical research. To do so scientifically, one must observe and analyze “the types of engagement of the knowing subject” (Van de Vijver, et al., 2005, p. 68) through which the researcher actively engages in the re-creation of the myriad different forms and processes in which life manifests itself.^18^

## Discussion and conclusion

The problem of the living organism lies in between the properties of objective matter and those of the subjective mind. While matter is inert and reacts to external forces in a mechanical way, mind is a source of an inner, seemingly autonomous, teleological activity. The problem with the organism is that it is perceived as matter but seems to act like mind, and this problem has led to almost endless discussion throughout the history of biology. However, neither reductionist approaches, which aim to explain life in terms of matter, nor vitalist theories, which postulate supernatural life forces, have been successful, and organicism, often seen as a third way between reductionism and dualism, is mostly descriptive but not explanatory.

Here, I suggest a solution to the organism-problem which draws on the Kantian notion that the knowing mind provides the conditions of knowability of any known object of experience (“object” in the broadest sense). Objects of experience, according to Kant, are being constituted by an active cognitive process. I argue that the life of an organism, i.e., its teleological, self-generating power, can be cognized within a co-constitutive process, in which the observer actively follows the developmental activity of an organism. This activity can be experienced by what Kant called an “inner sense” (Kant [1786], 2004, 4:467). The observation of this experience can be called *empirical cognition of the formative force of life*. This approach, therefore, overcomes attempts to reduce life to non-living matter and provides a solution to the central problem of vitalism by showing how the formative force of an organism can become an object of empirical observation.

The argument presented here rests on Kant’s analysis of the organism-problem. This may be criticized as an arbitrary restriction, since it has been argued that, e.g., Hans Jonas’ philosophy of the organism would be a better starting point (Weber & Varela, 2002). However, in my understanding Kant is central to the discussion because he went to the very root of the organism-problem, namely the conditions of its knowability. And from this root, as is argued here, an expanded view can be developed.

By demonstrating how the formative force of an organism can be observed, only the first half of the organism-problem is solved, i.e., the necessity to demonstrate the reality of a force of the living. The second half of the problem consists of the incompatibility of the living with a conception of nature in merely materialistic terms. Therefore, I suggest an expansion of the concept of nature to allow for intelligent forces of life. Since this must not be a speculative assumption, I draw on an argument by Van de Vijver and colleagues who suggested the existence of an “ontological space” of the living which exists in addition to the ontological space of the physical. This ontological space of the living is constituted in a co-determining encounter between the knowing subject and the known, living object.

It may be emphasized once again that the study of organisms requires two different modes of empirical access: *objectifying* observation of their physical, sensually perceptible properties and *realizing* observation of their processual and teleological, self-generating life-processes. While the first mode leads to cognition of the physical structures and physiological functions of an organism (which are subject to physico-chemical laws and therefore, to large extents, can be reproduced in a test tube), the second mode provides empirical access to the vital force which generated these structures and functions and to their teleological meaning (which are not physico-chemical and cannot be artificially created). The clear distinction between these two modes of cognition, which can and must complement each other, may lead to greater clarity of questions about the organism.

In summary, I argue that the eternal problem of understanding an organism—the question whether the existence of an organic formative force is merely a speculative assumption or an objective reality—can be solved. From this point of view, attempts to reduce life to purely material interactions are no longer necessary. However, the physico-chemical side of an organism can be seen in a new light, not as a cause, but as a condition and, above all, as an expression of the living, organic activity. I have called the observation of the co-constitutive formative activity “reading the book of nature”, and the physical and even the molecular structures and processes of an organism can also be “read” in this way. This can be achieved by observing the mental activities through which the different physiological, biochemical and molecular processes are conceived (Hueck, 2009). “Reading the book of nature” can also lead to an integrated concept of the organism as well as to new perspectives on evolution (Hueck, 2023).

The analysis and conclusion presented here also shed a new light on the reality of organismal autonomy and agency (Desmond & Huneman, 2020; Virenque & Mossio, 2024). It can be argued that the reality of these properties of an organism can be observed within a re-creating encounter in the same way as that of its organic, formative force. However, while the *developmental change* of organic shapes is being re-created within *active imagination*, and while the *teleology* of the process is being re-created by *concepts* of understanding, organic *autonomous agency* (the ability of organisms to “initiate their own changes” (Walsh, 2018, p. 176)) is being re-created within the ability of *willful action*.

It goes without saying that these considerations have many references to the ongoing discussion about Kant’s understanding of the cognition of an organism (e.g., Gambarotto & Nahas, 2022; Ginsborg, 2015a; Kreines, 2005), to the philosophy of the organic of Schelling and Hegel (Illetterati & Gambarotto, 2020), to Merleau-Ponty’s (Thompson, 2010) and Hans Jonas’ (Gambarotto, 2020) phenomenological approach, to Whitehead’s process ontology (Koutroufinis & Araujo, 2023; Meincke, 2023), and to aspects of vitalism (Donohue & Wolfe, 2023), which cannot be discussed here.

However, a specific relation to Goethe’s way of morphological thinking must be highlighted, because Goethe can be called the father of the approach elaborated here (Amrine, 1998; Bauer, 2023; Bockemühl, 1998; Brady 1987; Förster, 2012; Robbins, 2006). Gregory Rupik has recently discussed Goethe’s method of cognition of the forces and laws of an organism which exactly corresponds to the method described here (Rupik, 2024). The main aspect of this method, which Goethe called “delicate empiricism” (*zarte Empirie*) is, in Rupiks words, to “carefully, patiently, and gingerly [follow] nature where it leads” (ibid., p. 40). Thus, Goethe, who continuously reflected on his approach to nature (Naydler, 2000), claimed that “[i]f we wish to arrive at some living perception of nature we ourselves must remain as quick and flexible as nature and follow the example she gives” (Goethe [1817], 1995, p. 63). This is exactly what has been described here as an active and participatory engagement with the developing organism. Furthermore, Goethe reported on his experience of a “point at which the human mind can most closely approach the objects in their generality, bring them to itself, amalgamate with them (as we otherwise do in common empiricism) in a rational way, as it were” (Goethe [1798, 1893], 1965 ff., p. 870; my translation). “Rational amalgamation” of the human mind with the teleological self-generation force of an organism is achieved by the re-creative cognition of the living being. Finally, Goethe once claimed that “where object and subject meet, there is life” (Goethe [1827], 2000). This is probably the shortest possible expression of the solution to the organism-problem.

Therefore, as long as one applies only the Kantian mode of objectification to an organism, one might say with Goethe, “Whoever wants to recognize and describe something living,/First seeks to drive out the spirit;/Then he has the parts in his hand,/Missing, alas! only the spiritual [mental] bond.” (Goethe [1808], 1960 ff., p. 208; my translation). Applying the second mode of Goethean realization provides the “mental bond”. An interesting historical note is that both Kant’s *Critique of Judgment* and Goethe’s *Metamorphosis of Plants* (Goethe, [1790, 1817, 1831] 1965 ff.), in which he demonstrated his method, were both published at Easter 1790. While Kant analyzed the organism-problem in the most fundamental way, it was Goethe who showed in principle and practice how to solve it.

## Footnotes

1 See also the collection of essays on the concept of the organism in History and Philosophy of the Life Sciences, 2010.

2 The comparison of organisms with self-maintaining dissipative systems—hurricanes, candle flames, Bénard cells (Moreno & Mossio, 2015)—is not sufficient to explain how living organisms can emerge from material conditions, because dissipative systems do not generate offspring (Soto et al., 2016). Another attractive model which attempts to explain “minimal autonomous systems” by so-called “work-constraint cycles” (Kauffman & Clayton, 2006) faces the same problem. The litmus test for whether organisms can be explained in naturalistic terms would be the artificial creation of such minimal autonomous and replicating systems in a bottom-up approach, which, despite intensive experimental research (Hanczyc, 2020; Luisi, 2006), is still impossible (Chang et al., 2023; Porcar et al., 2011). Finally, the origin of life in a cosmos that is thought of as non-living remains an unresolved question (Sutherland, 2017; Preiner et al., 2020; Lane & Xavier, 2024).

3 Arguably, there can be no deeper root than this, because one can only analyze and discuss what one knows (knowing construed in the broadest sense).

4 In the *CJ*, Kant extensively discussed the concept of teleology, while he mentioned the “formative power” of organisms only in § 65 and—referring to Blumenbach—in § 81 (Goy, 2012). However, he argued that both criteria—teleological organization *and* self-generating power are necessary to distinguish organisms from mechanisms (Kant [1790], 2008, 5:373), because teleological organization is also found in mechanical devices, which, however, do not construct, nor repair or reproduce themselves (cf. Ginsborg, 2015b; Huneman, 2017).—Teleology and self-generation certainly do not represent a comprehensive description of the “properties of life” (cf. Rosslenbroich, 2023). However, other aspects of the organism such as metabolism, organizational closure, reproduction, development, individuality, autonomy, agency, adaptability, or evolvability, although being additional and intimately intertwined properties, presuppose or at least include teleological self-organization (Bich & Damiano, 2007; Ruiz-Mirazo & Moreno, 2012; Mossio & Bich, 2014; Walsh, 2015; Fulda, 2023; Nuño de La Rosa, 2023).

5 Matter, according to Kant’s definition in the *Metaphysical Foundations of Natural Science* (Kant [1786], 2004), is a “mere object of outer sense, [which] has no other determinations except those of external relations in space, and therefore undergoes no change except by motion” through external causes (Kant [1786], 2004, 4:543). In contrast, life is “the faculty of a substance to determine itself to act from an internal principle” (Kant [1786], 2004, AA 4:544). The internal activity of living beings, therefore, cannot be unified with a materialistic conception of nature. (For an extended analysis of Kant’s concept of matter cf. Friedman, 2015, and for life cf. Nunez, 2021.).

6 Such unification is attempted by theorists of autopoiesis. However, this theory has not been successful so far (cf. ftn. 2). A detailed discussion would exceed the scope of this paper; cf. also (Wolfe, 2010, 2024). Other authors have argued that in order to explain organisms we need to consider a different conception of nature, arguing for processual and emergentist metaphysics related to Alfred North Whitehead’s process ontology (Koutroufinis, 2024; Meincke, 2023; Segall, 2023), to Schelling’s *Naturphilosophie*, which views nature as inherently creative and self-organizing (Gambarotto & Nahas, 2023; Rupik, 2024), claiming “a cosmic predisposition to the formation of life” (Nagel, 2012), or even calling for a new “agent theory of the universe” (Walsh, 2018).

7 In *On the Use of Teleological Principles in Philosophy* (1788) Kant wrote that “indeed every anatomist as well as every physiologist actually starts from this concept” of a purposive whole (Kant [1788], 2012, 5:181).

8 *Experience* is understood by Kant as the cognitive unity of empirical intuitions with concepts of understanding (Wuerth, 2021, p. 183).

9 Shape is not often considered in discussions about organismal autopoiesis, autonomy, agency, etc., likely because it is assumed that Darwinism explains it (e.g. Huneman, 2017; Gambarotto & Nahas, 2022; however, cf. Toepfer, 2021). This important point must also be discussed in epistemological terms but this discussion would go far beyond the scope of this paper.

10 Imagination not understood as phantasy, but as mental representation.

11 The question of whether or not organic development can be considered as “goal-directed” has been discussed by several authors (Bich & Skillings, 2024; Minelli, 2011; Rupik, 2021; Silvestros, 2023). Here I simply assert that development proceeds from the bud to the leaf. Since the bud has the intrinsic potential to continuously develop into the leaf, the latter can be considered the “goal” of the developmental process. The specific configuration of the cellular, biochemical and molecular structures and processes in the bud only exists and occurs *because* they are the basis for further development into the leaf.

12 No living, teleologically organized whole can be conceived of without the concept of self-generation, and no organic self-generation is possible if it is not teleologically organized.

13 Kant considered the will to be a “faculty of acting according to ends” (Kant [1790], 2008, 5:370).

14 For example, the changes of the leaf shape in Fig. 1 appear to be governed by the organic law of polarity between expansion and contraction: While the leaf-blade expands, the petiole contracts. Several of such formative laws and their temporal structure in the developing organism have been described in detail (Bockemühl, 1998).

15 Van de Vijver & Noé wrote: “As the third (…) Critique can show, there is objectivity to the extent that an object is produced, in a very specific way, from within a living, contingently based, conditionality: an object is that which has been successfully ‘pushed outside’ of the sphere of the contingently based, largely implicit, practices of living subjects” (Van de Vijver & Noé, 2011, p. 98).

16 Kant himself felt the need to integrate his epistemological analysis of organic teleology into the systematic unity of nature. In his *Opus postumum* he developed the idea of “moving forces” inherent in matter which encompass the organizing forces of life (Förster, 2000).

17 This may seem contradictory to the Kantian notion of active constitution of physical “objects of experience” as described in Sect. 4. However, the slow transformation of one leaf-shape in Fig. 1 into another in one’s imagination certainly requires a much more intense activity than cognizing an individual shape as a “leaf” (cf. also discussion below on Goethe’s method of “delicate empiricism”).

18 The conscious observation of a participatory engagement with the processes of life can be called with an old expression: *Reading the book of nature*. Such “reading” can lead to astonishing, non-mechanistic insights even into the relation between the molecular and genetic processes of an organism and its life in general (Hueck, 2023).
